# Complex determinants of inappropriate use of antibiotics

**DOI:** 10.2471/BLT.17.199687

**Published:** 2018-01-10

**Authors:** Viroj Tangcharoensathien, Sunicha Chanvatik, Angkana Sommanustweechai

**Affiliations:** a International Health Policy Program, Ministry of Public Health, Nonthaburi 11000, Thailand.

## Demand and supply challenges

Inappropriate use of antibiotics in humans and agriculture is one of the drivers^1^ of the emergence of antimicrobial resistance. The prevalence of penicillin-resistant pneumococci, macrolide-resistant *Streptococcus pneumoniae* and *S. pyogenes* strongly correlates with total antibiotic use in outpatients.^2^ Such inappropriate use is the result of complex interactions between demand for and supply of antibiotics. The use of antibiotics in the retail sector, for example in pharmacies and drugstores, the health-care sector and in farms involves both professional and unqualified personnel from various sectors. These personnel operate within their country-specific regulatory environments and have different economic incentives.

In the retail sector, there are several challenges on the demand for antibiotics. Prevalence of non-prescription antibiotics is high in some countries, such as in Bangladesh, Brazil and Sudan. In these countries, the prevalence of antimicrobial resistance is higher in communities who use non-prescription antibiotics more frequently.^3^ Among Hispanic households in New York, United States of America (USA), self-medication with antibiotics is a common choice for fever, respiratory and gastrointestinal conditions.^4^ A systematic review of adult individuals in households of low- and middle-income countries covering 34 studies shows high prevalence (39%) of antimicrobial self-medication.^5^ Half of these studies focus on non-prescription antibiotics while the remainder is about the use of anti-malarial and other medicines. Main sources of self-medication antibiotics were found to be pharmacies, drugstores and leftover or borrowed drugs. Antibiotics sold in drugstores and provided by non-qualified personnel exacerbate inappropriate use. Common inappropriate use by households includes not completing the course or taking an insufficient dose, taking antibiotics for the wrong indications, such as viral infection and inflammation, and sharing antibiotics. The antibiotics most commonly used inappropriately to treat flu or common cold symptoms are ampicillin, tetracycline, metronidazole, ceftriaxone, kanamycin and cotrimoxazole.^5^

Health-care markets, understood as the interface between demand and supply, are imperfect, because professionals have more technical information than patients; this is also known as asymmetry of information.^6^ Health professionals who prescribe or dispense antibiotics, when motivated by financial incentives, can induce demand through the unnecessary use of antibiotics.

In South Asian countries, common challenges on the supply side in the retail sector^7^ are poor quality dispensing, particularly by unqualified providers, and inadequate labelling and counselling. Other challenges include insufficient clinical history taking and sale of antibiotics that have no proper dosage or are clinically inappropriate.^8^ Common sources of poor counselling are unqualified drug sellers, patients using old prescriptions and word of mouth from friends.

In the Australian health-care sector, there are gaps such as the prescribing knowledge among junior doctors, the lack of awareness on the limited use of restricted antibiotic classes^9^ and antibiotics are often prescribed in the absence of a laboratory sensitivity test. In Peru, poor knowledge of local resistance rates and profiles leads to inappropriate prescribing, and patient demand for antibiotics further complicate the problem.^10^ A study across 17 European countries shows that a 1% increase in doctor-to-population density is associated with 0.52% to 0.86% increase in outpatient use of antibiotics, and that a fee-for-service incentivises higher use than the capitation payment method.^11^

Foodborne transmission of antimicrobial-resistant bacteria from animals to people is well documented.^12^^,^^13^ For instance, the emergence of fluoroquinolone-resistant Campylobacter has been attributed to the consumption of poultry meat in the USA which has led to banning the use of fluoroquinolones in poultry.^14^ In the agricultural sector, veterinarian prescriptions in some countries are influenced by their training, published literature, experience, sensitivity tests, risk of resistance and ease of administration.^15^ Another study carried out in 25 European countries showed that about a half (44.3%) of the 3004 veterinarians who participated in the study seldom collect samples for laboratory bacterial diagnosis before prescription, while 37.8% frequently undertook the test.^16^ In Cambodia, a study has shown that farmers have limited understanding of the action and indication for antibiotics.^17^ Farmers also had a low level of risk perception about the implications of antimicrobial resistance in livestock and a lack of concern about potential links between antibiotic use in farming and resistance in humans. In contrast, German farmers have a high level of awareness on the links between antimicrobial resistance in humans and animals and the need for prudent antibiotic prescribing.^18^ In some countries, the high cost of animal feed that contains antibiotics pushes farmers to use active pharmaceutical ingredients mixes with animal feed, with no quality control over the homogeneity of antibiotics in the feed.

## Effective interventions

**Fig. 1:**
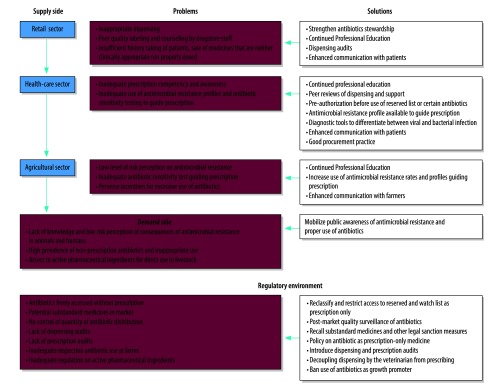
Problems and solutions to inappropriate use of antibiotics

Fig. 1 presents policy interventions on the demand and supply sides that address the challenges of inappropriate use of antibiotics in the retail, health and agricultural sectors.

In countries where antibiotics are freely available without prescription, the regulatory environment should be reformed. This reform could be done through a reclassification of antibiotics, whereby certain antibiotics on the watch list, which includes those antibiotics that are recommended as a first or second choice treatment for some infections, and all the reserved items,^19^ are restricted. This means they would only be used in the most severe circumstances, when all other alternatives have failed, for instance to treat life-threatening infections due to multidrug-resistant bacteria. Ensuring quality of medicines through reinforcing regulations and establishing good procurement practices and post-market surveillance of medicine quality is essential to containing antimicrobial resistance, as demonstrated in the successful regulation of veterinary medicines in the Republic of Korea.^20^

The challenges of the availability of non-prescription antibiotics in the retail sector can be addressed by introducing electronic dispensing audits to control the use of unauthorized reserved items and the excessive use of the watch list. Hospital prescription audits, either by professional peers such as the Swedish strategic programme against antibiotic resistance (Strama)^21^ or by an official regulatory body should also be developed as critical entry points for improved antibiotic stewardship. Manual sample audits can be applied in countries that have difficulties in introducing electronic audits and become a platform for future electronic audits. Positive incentives and punitive measures should be introduced by regulators or hospitals following the antibiotic stewardship programme to achieve prudent use, while enhanced communication with patients could contribute to minimizing unnecessary demand for antibiotics.

In the health-care sector, inappropriate use by professionals can be reduced with education and training, peer review and support, preauthorization of certain antibiotics by experts on infectious disease, better diagnostic tools, and use of resistance profiles. These profiles would guide prescriptions where antimicrobial sensitivity tests of bacteria are not available, for example in smaller hospitals. Excessive use of antibiotics can be addressed by controlling the financial incentives linked to antibiotics and by providing continued professional education.

In the agricultural sector, inappropriate and excessive use can be reduced by: delinking the financial gains from prescribing and dispensing veterinary antibiotics; banning the use of antibiotics as growth promoters; auditing farm veterinarians; and providing training on the use of different classes of antibiotics, particularly the reserved list. Literature suggests that pressure from farmers to use antibiotics and the financial gains from antibiotics use affects prescribing behaviour. For instance, in Denmark, interventions that led to a significant reduction of antibiotic consumption included delinking veterinarian prescribing and dispensing, and decreasing the maximum profit that veterinarians obtain from antibiotic sales from 25% to 5%.^22^

## Monitoring gaps

Tools are available for monitoring knowledge about antibiotics and awareness of resistance in the general population, such as the European Commission’s special Euro-barometers 338,^23^ 445,^24^ flash Eurobarometer^25^ and the World Health Organization’s (WHO) special survey in 12 countries.^26^ Population surveys apply different methods that vary according to country context and survey resources. Reviews showed that the questionnaire used in these population surveys usually cover self-reported use of antibiotics over a period of 6–12 months where prevalence, profile of non-prescription antibiotics and sources of providers can be established.

To survey farmers and veterinarians, the European Food Safety Authority’s survey covers four sets of questions: (i) understanding the relationship of antibiotic use and antimicrobial resistance in animals and human health; (ii) risk perceptions of developing such resistance in animal farming; (iii) reasons and rationales underpinning risk perceptions; and (iv) channels that influence the perceptions and practices of veterinarians and farmers.^27^

We have identified the following gaps in monitoring. Monitoring prescription knowledge and practice, awareness and incentives for prescribing antibiotics among physicians and veterinarians is sporadic, most of these studies are research projects, less frequently, small-scale and not a national monitoring system. Yet, professional prescribing competency, practice, knowledge and awareness of local antimicrobial resistance profiles need to be monitored. Identified gaps in prescribing competency can guide the contents of in-service continued professional education.

In the agricultural sector, the use of antibiotics in aquatic food production and plants needs to be monitored, for example the treatment of greening disease in citrus trees.^28^ In the food-chain, as one of the major transmission pathways of antimicrobial resistance, knowledge, practice and awareness among food-handling personnel needs to be surveyed. Among farmers, knowledge and practice of the withdrawal period, the minimum duration from administration of antibiotics until slaughter of an animal, should guide specific messages for practice modifications. The European Food and Safety Authority’s tool is useful and can be applied to monitor the livestock sector.

The relative size of the volume of antibiotics consumed at the retail, primary care, hospital and agricultural sectors, as well as the prevalence of inappropriate use in these sectors will identify areas that need more policy focus.

## Recommendations

Addressing the inappropriate use of antibiotics requires multifaceted corrective measures that should be guided by monitoring findings. Reclassification of and limited access to reserved and watch-list antibiotics and post-marketing quality surveillance would ensure that antibiotics remain efficient. In the retail sector, where antibiotics are at highest risk of being inappropriately used, policy needs to focus on keeping dispensing records that can be audited by professional peers. The hospital sector should improve stewardship through support and peer reviews, requiring preauthorization for certain antibiotics and ensuring that antimicrobial resistance profiles guide clinical decisions before and after the results of sensitivity tests. Recommended actions for the agricultural sector are banning the use of antibiotics as growth promoters, delinking prescribing from dispensing and carrying out audits. Last, in-service continuing professional development training is essential to all sectors.
